# Induction Chemotherapy Combined With Intensity-Modulated Radiotherapy for 129 Nasopharyngeal Carcinoma Patients With Synchronous Metastases: A Retrospective Study

**DOI:** 10.3389/fonc.2021.654871

**Published:** 2021-05-21

**Authors:** Mengshan Ni, Lijun Geng, Fangfang Kong, Chengrun Du, Ruiping Zhai, Yingchen Lyu, Chaosu Hu, Hongmei Ying

**Affiliations:** ^1^ Department of Radiation Oncology, Fudan University Shanghai Cancer Center, Shanghai, China; ^2^ Department of Oncology, Shanghai Medical College, Fudan University, Shanghai, China; ^3^ Shanghai Key Laboratory of Radiation Oncology, Shanghai, China

**Keywords:** NPC, synchronous metastasis, survival rate, IMRT, induction chemotherapy, prognosis

## Abstract

**Objective:**

To analyze the therapeutic effect and prognostic factors of nasopharyngeal carcinoma (NPC) patients with distant metastases at initial diagnosis receiving induction chemotherapy with intensity-modulated radiotherapy (IMRT).

**Methods:**

A total of 129 patients who underwent platinum-based induction chemotherapy followed by definitive IMRT with or without concurrent or adjuvant chemotherapy for newly diagnosed distant metastatic NPC in our center between March 2008 and November 2018 were retrospectively analyzed. 41 patients underwent local therapy for metastatic sites. Kaplan-Meier method was used to estimate survival rates, Log-rank test and Cox proportional hazards model were used to figure out independent prognostic factors of overall survival (OS).

**Results:**

A total of 66 patients had been dead (median follow-up time, 51.5 months). The median overall survival (OS) time was 54.2 months (range, 7-136 months), and the 1-year, 2-year, 3-year, 5-year overall survival rates were 88.0%,71.0%,58.0%, and 47.0%. Multivariate analysis found that the factors correlated with poor overall survival were pre-treatment serum lactate dehydrogenase (SLDH) >180U/L, chemotherapy cycles<4, and M1 stage subdivision (M1b, single hepatic metastasis and/or multiple metastases excluding the liver; and M1c, multiple hepatic metastases). The 5-year OS rates for M1a, M1b and M1c were 62.6%,40.4% and 0%, respectively.

**Conclusion:**

Platinum-containing induction chemotherapy combined with IMRT seemed to be advantageous to prolong survival for some NPC patients with synchronous metastases at initial diagnosis. The independent factors to prognosticate OS were pre-treatment SLDH, number of chemotherapy cycles, and M1 subcategories. Prospective clinical trials are needed to confirm the result.

## Introduction

Nasopharyngeal carcinoma is an Epstein-Barr virus (EBV) related epithelial malignant tumor that occurs commonly in South-Eastern Asia and Southern China (1). The occurrence of NPC in China is notably high, especially in Guangdong, Hainan, Hunan, and Fujian provinces (2). Among newly diagnosed NPC patients, the majority of them were locally advanced, while nearly 4% cases with synchronous distant metastasis (3–5). Although significant improvement has been achieved in survival benefits for patients with locally advanced NPC, the overall survival is remarkably poor once metastasis is diagnosed. Furthermore, it has been reported that patients with synchronous metastases had shorter survival compared with those with metachronous metastases (6).

At present, there is no high-level evidence-based medical recommendation for the therapeutic strategy of patients with newly diagnosed metastatic NPC. Systemic chemotherapy has been the most frequently used treatment approach for palliative intent. Whether to perform radiotherapy on primary tumor lesions of these patients remains controversial. Recently, an increasing amount of clinical studies (7–10) suggest that chemoradiotherapy could improve the OS rate of patients with initially metastatic NPC. A retrospective study (8) showed that those who received chemoradiotherapy have a higher 3-year OS rate compared with those who only received chemotherapy(48.2% *vs.* 12.4%, p<0.001). Nevertheless, the combinations of chemotherapy sequences related to radiotherapy were diversified in these researches, including induction, concurrent, adjuvant, or other combination. In addition, the radiation techniques used in these studies varied differently, including two-dimensional conventional radiotherapy (2D-CRT), three-dimensional conformal radiotherapy (3D-CRT), and IMRT. These differences may have a profound impact on therapeutic efficacy. Meanwhile, the M1 stage is an “all-embracing” categorization, which encompasses the whole range of metastatic disease from oligo-metastases to disseminated metastases. Considering the great heterogeneity, patients in the M1 stage do not always achieve similar efficacy or have the same failure patterns. In the oligo-metastases phase, patients may receive more aggressive treatment. To optimize the management of patients with metastatic NPC, some scholars have conducted to subdivide the M1 stage (4, 11–14). A retrospective study (13) redivided the M1 stage into three categories: M1a, single metastasis except for the liver; M1b, single hepatic metastasis and/or multiple metastases not including the liver; and M1c, multiple hepatic metastases. The 3-year OS rates were 62.1%, 36.1%, 17.9%, correspondingly.

On the basis of the above-mentioned M1 subcategories, we performed this study to analyze the outcome of synchronous metastatic NPC patients treated with induction chemotherapy in advance of IMRT to the primary and cervical disease, with or without local therapy to metastatic foci, with the intention to investigate the therapeutic approaches and prognostic factors for patients with metastatic NPC.

## Patients and Methods

### Patients and Selection Criteria

A total of 129 newly diagnosed NPC patients presenting with synchronous metastases treated with chemoradiotherapy at Fudan University Shanghai Cancer Center between March 2008 and November 2018 were enrolled in this retrospective study. The inclusion criteria were (1): Be at least 18 years old; (2) Pathologically confirmed nasopharyngeal carcinoma; (3) Eastern Cooperative Oncology Group (ECOG) performance status (PS) score ≤ 1; (4) Synchronous distant metastases at initial diagnosis. Metastases were confirmed *via* pathological biopsy or at least two kinds of imaging examinations; (5) Receiving induction chemotherapy and IMRT to the primary lesion; (6) Complete clinical data and follow-up. TNM stage of these NPC patients was classified according to the eighth edition of the American Joint Committee on Cancer (AJCC) staging system. M1 stage was categorized in accordance with the retrospective study as mentioned above. The clinical features of these 129 patients are elaborated in **Table 1**.

**Table 1:** Clinical characteristics.

Characteristic	Number of patients	Percentage (%)
Age years		
≤ 50	66	51.2
>50	63	48.8
Sex		
male	98	76.0
female	31	24.0
Tumor classification^a^		
T1	20	15.5
T2	22	17.0
T3	61	47.3
T4	26	20.2
Nodal classification^a^		
N0	3	2.3
N1	16	12.4
N2	45	34.9
N3	65	50.4
Sites of metastasis		
Bone	100	77.5
Single/Multiple	37/63	28.7/48.8
Lung	25	19.4
Single/Multiple	6/19	4.7/14.7
Liver	16	12.4
Single/Multiple	7/9	5.4/7.0
Other Organs	1	0.8
Single/Multiple	1/0	0.8/0
M1subdivision^b^		
M1a	40	31.0
M1b	80	62.0
M1c	9	7.0
Serum lactate dehydrogenase (SLDH) (U/L)		
≤180	62	48.0
>180	67	52.0

a Tumor-node-metastasis staging system proposed by the American Joint Committee on Cancer (8^th^ edition).

b M1 stage subdivision; M1a, single metastasis in any location other than the liver; M1b, single hepatic metastasis and/or multiple metastases excluding the liver; M1c, multiple hepatic metastases.

The current research was endorsed by the Institutional Review Board of Fudan University Shanghai Cancer Center. The research was implemented in conformity with our institutional criterion and the 1964 Declaration of Helsinki and its revisions.

### Pre-Treatment Assessment

All these NPC patients accomplished pre-treatment workup comprising medical history, physical examinations, nasopharyngofiberoscope with biopsy, MRI or contrast-enhanced CT scan of the nasopharynx and neck regions, chest CT scans, abdominal ultrasound, whole-body bone scan by Emission CT, whole-body scan by positron emission tomography/computed tomography (PET/CT) if necessary, blood routine, serum biochemical profiles, common infectious disease screening, electrocardiogram. Patients were evaluated in compliance with the eighth edition of AJCC TNM stages.

### Treatment Protocol

All the patients underwent platinum-based induction chemotherapy with or without concurrent or adjuvant chemotherapy. The undermentioned regimens were commonly used for induction and adjuvant chemotherapy: TP (docetaxel 75 mg/m ^2^/day, day 1 + cisplatin 25 mg/m ^2^/day, day 1-3), GP (gemcitabine 1g/m ^2^/day, day 1,day 8 + cisplatin 25mg/m ^2^/day, day 1-3) or TPF (docetaxel 75 mg/m ^2^/day, day 1 +cisplatin 25 mg/m^2^/day, day 1-3 + 5-fluorouracil 0.5g/m ^2^/day, day 1-3) regimen. Induction and adjuvant chemotherapy were dispensed every 3 weeks. Simultaneous chemotherapy was comprised of cisplatin (80 mg/m ^2^), which was administered every 3 weeks for 2-3 cycles.

All patients were treated with external-beam radiotherapy performed with IMRT after the completion of induction chemotherapy. Patients were immobilized in the supine position with thermoplastic casts. T1-weighted images in the transverse plane of enhanced MRI would be fused with the planning CT images for gross tumor volume (GTV) delineation. The prescribed dose was 60–70.4 Gy to the planning target volume (PTV) of the gross tumor volume of the nasopharynx and metastatic neck lymph nodes in 30–35 fractions, 60 Gy to the PTV of the high-risk region, and 54 Gy to the PTV of the low-risk region. Some patients received an additional boost of radiation (PTV-Boost) for local residual lesions.

Furthermore, 41(31.8%) patients underwent local treatment to the metastases, which include 32(24.8%) patients who received radiotherapy to the bone metastases (30–40 Gy/10–20 fractions), 4(3.1%) patients received surgery for vertebral metastases. 6(4.7%) patients received interventional or radiofrequency ablation for hepatic metastases, and 5(3.9%) patients underwent surgery, gamma knife therapy, microwave ablation therapy, or SBRT for lung lesions.

### Patient Evaluation and Follow-Up

All the patients were examined weekly during radiotherapy. Intravenous or oral nutritional support was given to patients with treatment-induced oral mucositis (grade ≥II).

Following the completion of IMRT, patients who achieved a complete response were followed up every 3 months during the first 2 years, every 6 months in the third to fifth year, and then annually thereafter. Indirect or direct nasopharyngoscopy was routinely performed during each follow-up. MRI or CT of the nasopharynx, chest CT scan, ultrasound of the abdomen, and radiologic imaging of the distant metastasis were carried out in the third month and every 6 months afterward. For patients who did not achieve a complete response, the follow-up period would be adjusted appropriately.

### Statistical Analysis

Data analysis was performed utilizing the SPSS version 21.0 (IBM, Armonk, NY, USA). Treatment-related toxicities were recorded in accordance with Common Terminology Criteria for Adverse Events (version 3.0). The primary endpoint of this study was overall survival (OS). The definition of OS was the length of time from the date of diagnosis to the date of death due to any cause or the date of the last follow-up for patients still alive. OS was estimated by the Kaplan–Meier method. ROC curve was performed to evaluate the apposite cutoff point for SLDH level before treatment. Patients’ clinical characteristics (sex, age, SLDH), tumor features (T classification, N classification, M subdivision, number of metastatic foci), and therapeutic strategies (number of total chemotherapy cycles, the dose of radiotherapy, local therapy to metastases) were analyzed *via* log-rank test. Categorical variables of treatment modes for patients with different M1 stage subdivision were compared using χ^2^-test (or Fischer’s exact test, if indicated). Univariate and multivariate analyses were performed by use of the Cox proportional hazards model. Variables associated with OS were identified *via* multivariate analyses. Both forward and backward stepwise procedures were performed. Any result with a two-tailed P value<0.05 was considered statistically significant.

## Results

### Survival

The characteristics of the 129 patients are presented in **Tables 1** and **2**. All the patients were histologically confirmed nasopharyngeal carcinoma (WHO type II/III). There were 9(7.0%) patients with pathologically confirmed metastases and 120(93.0%) cases of distant metastases were diagnosed through imaging examinations. The median follow-up time for the whole group was 51.5 months. At the time of the last follow-up, 66 patients had been dead. The median OS time was 54.2 months (range, 7-136 months), and the 1-year, 2-year, 3-year,5-year overall survival rates were 88%,71%,58%,47%, respectively. Of the 129 patients,11(8.5%) patients developed local regional failure at the primary lesion, retropharyngeal lymph nodes, or cervical lymph nodes, and 67(51.9%) patients developed new metastatic lesions after IMRT.

**Table 2:** Treatment characteristics.

Characteristics	Number of patients	Percentage (%)
Radiation dose of the nasopharynx and neck(Gy)		
<66	34	26.4
≥66	95	73.6
Local treatment of metastatic disease		
Yes	41	31.8
radiotherapy	35	27.1
surgery	5	3.9
others	6	4.7
No	88	68.2
Total chemotherapy cycles		
4	9	7.0
4-6	92	71.3
6	28	21.7
Concurrent chemotherapy		
Yes	7	5.4
No	122	94.6
Adjuvant chemotherapy		
Yes	30	23.3
No	99	76.7
Anti-EGFR monoclonal antibodies		
Yes	9	7.0
No	120	93.0
New metastatic lesions		
Yes	67	51.9
No	62	48.1
Local recurrence		
Yes	11	8.5
No	118	91.5

The median induction chemotherapy cycle was 6(range 2-12). 109(84.5%) patients were treated with doublet regimens, 20 (15.5%) patients received triplet regimens. 30(23.3%) patients received adjuvant chemotherapy. The median number of full-course chemotherapy cycles for 129 patients was 6 (range 2–19). 7(5.4%) patients underwent simultaneous chemotherapy. All patients achieved a partial response to the induction chemotherapy. In this study, 95(73.6%) patients received radical radiotherapy (≥66 Gy). The remaining 34(26.4%) patients were treated with a dose less than 66 Gy. The median dose was 66 Gy (range, 59.4–75.4 Gy). 9(7.0%) patients received target therapy(cetuximab or nimotuzumab) contemporaneously with radiotherapy. Among the forty-one patients who received local treatment of metastatic sites, ten patients were classified as stage M1a, twenty-eight patients were categorized as stage M1b, and three patients were classified as M1c.

22(17.0%) patients who failed the front-line treatment underwent salvage treatment: 16(12.4%) patients received TP or GP regimens and the remaining patients received single-agent chemotherapy (capecitabine or gemcitabine or tegafur, gimeracil, and oteracil potassium capsules). 5(3.9%) patients received PD-1/PD-L1 immunotherapy after IMRT.

**Table 3** summarized the results of χ^2^ test analysis of M1a, M1b, M1c patients distributed in different treatment modes. In the current study, there were no significant differences in the choice of treatment methods made by clinicians between patients vary in M1 stage subgroups. Furthermore, due to the limited number of patients, this study did not conduct further treatment-related survival analysis for patients with different M1 substages.

**Table 3:** χ^2^ -test analysis of the patient distributions in different treatment modes.

M1subdivision^a^	Treatment characteristics				X²	P
	**Radiation dose of the nasopharynx and neck(Gy)**					
	≥66 (%)		≥66 (%)			
M1a	10(7.7)		30(23.3)		3.957	0.143
M1b	19(14.7)		61 (47.3)			
M1c	5(3.9)		4(3.1)			
	**Local treatment of metastatic disease**					
	Yes (%)		No (%)			
M1a	10(7.7)		30(23.3)		1.284	0.536
M1b	28(21.7)		52(40.3)			
M1c	3(2.3)		6(4.7)			
	**Total chemotherapy cycles**					
	4 (%)	4-6 (%)		>6 (%)		
M1a	4(3.1)	31(24.0)		5(3.9)	7.342	0.092
M1b	5(3.9)	56(43.4)		19(14.7)		
M1c	0(0)	4(3.1)		5(3.9)		

a M1 stage subdivision; M1a, single metastasis in any location other than the liver; M1b, single hepatic metastasis and/or multiple metastases excluding the liver; M1c, multiple hepatic metastases.

### Toxicities

In this study, none of the patients died of treatment-related toxicity. Grade III-IV hematological toxic effects were mainly leukocytopenia and neutropenia, which appeared in 69(53.5%) of all patients. The most significant non-hematological acute toxicities after radiotherapy were radiation mucositis and radiation dermatitis. 18(14%) and 2 (1.6%) of all patients suffered from grade III radiation mucositis and skin reaction, respectively. No grade IV non-hematological adverse effects were recorded (more details in **Table 4**).

**Table 4:** Summary of treatment-related toxicity.

	Induction chemotherapy (n=129)			Radiotherapy or CCRT^a^ (n=129)			Adjuvant chemotherapy (n=30)		
	Grade 0 or 1	Grade 2	Grade 3 or 4	Grade 0 or 1	Grade 2	Grade 3 or 4	Grade 0 or 1	Grade 2	Grade 3 or 4
**Acute hematologic toxicity**									
Anemia	103(79.9%)	23(17.8%)	3(2.3%)	126(97.7%)	3(2.3%)	0(0%)	21(70%)	5(16.7%)	4(13.3%)
Thrombocytopenia	111(86.0%)	10(7.8%)	8(6.2%)	115(89.1%)	14(10.9%)	0(0%)	23(76.7%)	4(13.3%)	3(10%)
Neutropenia	55(42.7%)	27(20.9%)	47(36.4%)	122(94.6%)	4(3.1%)	3(2.3%)	19(63.3%)	6(20.0%)	5(16.7%)
Leukopenia	64(49.6%)	35(27.1%)	30(23.3%)	111(86.0%)	14(10.9%)	4(3.1%)	14(46.7%)	10(33.3%)	6(20.0%)
**Acute non-hematologic toxicity**									
Radiation dermatitis	NA	NA	NA	121(93.8%)	6(4.6%)	2(1.6%)	NA	NA	NA
Radiation Mucositis	NA	NA	NA	64(49.6%)	47(36.4%)	18(14.0%)	NA	NA	NA

NA, not applicable.

a CCRT: IMRT combined with concurrent cisplatin or concurrent anti-EGFR monoclonal antibodies.

### Receiver Operating Characteristic Curve Analysis

The cutoff point for pre-treatment SLDH was determined as 180 U/L, which was obtained *via* ROC curve analysis. The area under the ROC curve was 0.65. There were 62 (48.0%) patients with pre-treatment SLDH ≤ 180 U/L and 67 (52.0%) patients above this point. The sensitivity and specificity of this cutoff point were 68.2% and 60.3% respectively.

### Univariate and Multivariate Analysis

Univariate analysis (**Table 5**) revealed that the negative prognostic factors for OS were including SLDH>180U/L (P=0.008), multiple bone metastases (P=0.012), M1 stage subdivision (P < 0.001), radiation dose<66 Gy (P=0.017),chemotherapy cycles<4 or>6 (P = 0.017), and the occurrence of new metastatic lesions (P=0.017). Multivariate analysis was performed on the potential negative prognostic factors, which were concluded through univariate analysis (**Table 6**). The result indicated that the significant negative prognostic factors were SLDH>180U/L (HR = 1.931, P = 0.015), M1 stage subdivision (M1b, HR = 1.851, P=0.047; M1c, HR = 5.632, P=0.001), Chemotherapy cycles<4 (HR =2.870, P, = 0.020).

**Table 5:** Univariate analysis of variables correlated with overall survival.

Prognostic factors	3y-OS (%)	5y-OS (%)	HR (95%CI)	P
Age(years)				
≤ 50	61.5	42.0	Baseline	
>50	57.6	47.8	0.898(0.548-1.472)	0.669
Sex				
female	75.6	54.0	Baseline	
male	54.3	42.5	1.256(0.713-2.211)	0.430
Tumor classification^a^				0.534
T1	69.8	27.0	Baseline	
T2	56.1	49.1	1.285(0.503-3.287)	0.600
T3	53.3	37.3	1.737(0.768-3.932)	0.185
T4	64.8	45.4	1.423(0.565-3.581)	0.454
Nodal classification^a^				0.547
N0	16.8	33.3	Baseline	
N1	56.9	60.0	0.701(0.143-3.437)	0.662
N2	63.8	50.8	0.814(0.189-3.513)	0.783
N3	56.5	35.1	1.141(0.263-4.959)	0.860
Sites of metastasis				
bone				
single	72.2	58.9	Baseline	
multiple	46.2	31.5	2.236(1.194-4.185)	0.012
lung				
single	75.0	75.0	Baseline	
multiple	87.4	55.0	1.484(0.173-12.727)	0.719
liver				
single	47.2	28.6	Baseline	
multiple	16.7	17.0	1.711(0.501-5.844)	0.392
M1subdivision^b^				0.000
M1a	71.6	62.6	Baseline	
M1b	59.3	40.4	1.989(1.089-3.634)	0.025
M1c	0	0	7.793(3.028-20.056)	0.000
Pre-treatment SLDH (U/L)				
≤180	71.8	60.9	Baseline	
>180	48.8	35.2	2.013(1.203-3.368)	0.008
Radiation dose(Gy)				
<66	32.8	29.5	Baseline	
≥66	69.1	49.9	0.544(0.330-0.896)	0.017
Local treatment of metastatic disease				
Yes	48.0	37.8	Baseline	
No	65.3	58.9	0.792(0.479-1.311)	0.365
Total chemotherapy cycles				0.017
4-6	64.3	53.3	Baseline	
<4	33.3	22.2	2.205(0.927-5.240)	0.074
>6	41.4	12.9	2.106(1.192-3.719)	0.010
Concurrent chemotherapy				
Yes	14.3	0	Baseline	
No	62.9	47.5	0.388(0.165-0.914)	0.030
Adjuvant chemotherapy				
Yes	56.6	34.0	Baseline	
No	59.4	47.5	0.906(0.507-1.617)	0.738
New metastatic lesions				
Yes	49.3	33.9	Baseline	
No	72.0	59.9	0.536(0.322-0.892)	0.017
Local recurrence				
Yes	68.2	54.5	Baseline	
No	58.3	44.1	1.213(0.570-2.582)	0.616

HR, hazard ratio; 95% CI, 95% confidence interval; SLDH, serum lactate dehydrogenase; 3y-OS,3- year overall survival rate; 5y-OS,5- year overall survival rate.

a Tumor-node-metastasis staging system proposed by the American Joint Committee on Cancer (8^th^ edition).

b M1 stage subdivision; M1a, single metastasis in any location other than the liver; M1b, single hepatic metastasis and/or multiple metastases excluding the liver; M1c, multiple hepatic metastases.

**Table 6:** Multivariate analysis of variables correlated with overall survival.

Prognostic factors	HR (95%CI)	P
Total chemotherapy cycles		0.034
4-6	baseline	
<4	2.870(1.183-6.965)	0.020
>6	1.612(0.880-2.954)	0.122
SLDH (U/L)		
≤180	baseline	
>180	1.931(1.134-3.287)	0.015
M1subdivision^a^		0.003
M1a	baseline	
M1b	1.851(1.009-3.396)	0.047
M1c	5.632(2.074-15.254)	0.001

a M1 stage subdivision; M1a, single metastasis in any location other than the liver; M1b, single hepatic metastasis and/or multiple metastases excluding the liver; M1c, multiple hepatic metastases.

The 5-year OS rate for patients with multiple bone metastases was 31.5%, notably poorer than patients with single bone metastasis (58.9%). Patients without new metastatic lesions had better 5-year OS (59.9%) than patients who had progressive disease (33.9%). Compared to the patients with normal serum LDH level, those with high serum LDH level had a distinctly worse survival (5-year OS: 60.9% *vs.* 35.2%). The 5-year OS rates for patients who received radiation dose≥66 Gy, <66 Gy were 49.9% and 29.5%, respectively.

**Figure 1:**
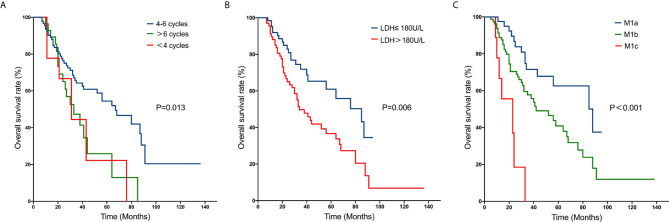
Kaplan–Meier curves of overall survival rates in patients with metastatic nasopharyngeal carcinoma: number of cycles of chemotherapy **(A)**; pre-treatment serum LDH level **(B)**; M1 stage subdivision **(C)**.

M1 stage subdivision was remarkably correlated with survival. The 5-year OS rate for M1a was 62.6% compared to 40.4% for M1b and 0% for M1c. The survival curves are shown in **Figure 1**. Furthermore, patients who were receiving 4 to 6 cycles of chemotherapy had a better 5-year OS rate than those receiving chemotherapy cycles<4 (53.3% *vs.* 22.2%).

## Discussion

Previous studies have confirmed that systemic chemotherapy can benefit patients with distant metastasis of nasopharyngeal carcinoma, and chemotherapy is considered to be the only possible treatment option. Some studies indicated that platinum-based double or triple chemotherapy regimens have high response rates in NPC patients and are currently the most widely used regimens (15–17). The NCCN guidelines also propose platinum-based combination chemotherapy or clinical trial as the first-line treatment for patients with newly diagnosed AJCC stage IVb NPC. Furthermore, the number of chemotherapy cycles is an independent factor associated with survival. Fandi et al. (18) reported a retrospective study including 20 metastatic NPC patients with long-term disease-free survival, the results demonstrated that at least 6 cycles of chemotherapy were required. A retrospective study (8) demonstrated that the OS of NPC patients treated with 4 cycles of chemotherapy was superior to those with <4 cycles. In the present study, the 5-year OS rate was 53.3% for patients who received 4 to 6 cycles, compared to 22.2% for patients who received <4 cycles. However, no obvious difference was observed between patients who received >6 cycles and the other two groups by the multivariate analysis, which may be relevant to the selection bias. The result is compatible with previous reports (8, 19). As the prolongation of the chemotherapy cycle can lead to drug resistance of tumor cells, it is recommended that metastatic NPC patients should receive 4-6 cycles of chemotherapy.

Radiation therapy is the cornerstone of the treatment strategies for patients with non-metastatic NPC. Due to the improvement in radiation techniques, intensity-modulated radiotherapy of nasopharynx and neck for NPC patients contributes to a good local control rate (20–22). However, its role in patients with initially diagnosed distant metastasis remains controversial. Yeh et al. (23) reported that the 2-year OS rate for patients with metastatic NPC who only received chemotherapy was 10.0% compared to 24.0% when they received radiotherapy alone. The study also inferred that the local control of the original lesions could have conspicuously promoted NPC patients’ life quality by reducing the occurrence of hemorrhage, necrosis, and severe headaches. Zeng et al. (8) reported that the 3-year OS rate of patients who received chemotherapy followed by radiotherapy was 48.2%, remarkably greater than those who only received chemotherapy (12.4%). Chen et al. (24) found that patients treated with chemoradiotherapy had an appreciably longer survival when compared with patients who underwent either radiotherapy alone or systemic chemotherapy alone. It is noteworthy that the first multicenter phase 3 randomized clinical trial (25) evaluating the efficacy of chemotherapy with or without locoregional IMRT in patients with synchronous metastatic NPC was reported in 2020. It showed that the 2-year OS rate and 2-year PFS rate in the chemotherapy combined with radiotherapy group were 76.4% and 35% respectively, compared with 54.5% and 3.6% in the chemotherapy alone group. In the current study, all the newly diagnosed metastatic NPC patients received induction chemotherapy followed by IMRT, the 5-year OS rate was 47%, obviously higher than the studies mentioned above. All these results suggest that locoregional radiotherapy of the primary lesions with IMRT help prolong the survival of NPC patients. Although efficacy differences of radiation dose on survival were only found in univariate analysis, a number of studies have confirmed that high-dose radiotherapy (≥ 65 Gy) of head and neck lesions can significantly prolong survival (24–26). Meanwhile, some studies indicated the sequence of combining chemotherapy and radiotherapy, including induction, simultaneous and adjuvant chemotherapy, may have different impacts on treatment results. Chen et al. (24) indicated that induction chemotherapy had relevance to the better survival rate, whereas concurrent and adjuvant chemotherapy, had not.

In the present study, the local treatment of metastatic lesions is not an independent prognostic factor affecting the overall survival of NPC patients with distant metastasis (P=0.365). Some studies also showed that local treatment of metastatic lesions was not strikingly associated with OS (4, 19, 24). Nevertheless, there is no therapeutic benefit in patients who underwent local treatment of metastatic sites, this might be rationalized by the fact that patients who received local treatment of metastatic sites in our department were mainly due to notable local symptoms and treatment wishes of patients.

The anatomic extent of metastasis was reported to associate with the prognosis of metastatic NPC patients. In a retrospective study including 263 patients with metastatic NPC, the 5-year OS rate was 4.8% for patients with multiple-organ metastases, while approximately 26.7% for patients with single-organ metastasis (19). In the study of 234 patients with newly diagnosed NPC with distant metastasis, patients with single metastasis also demonstrated superior survival benefits. The 3-year OS rate was 65.8% for those patients, compared to only 25.9% for patients with multiple metastases (8). In the meantime, this study also founded that liver metastasis was related to poor survival. Zeng et al. (8) reported that for patients with liver metastasis, the 3-year OS rate was only 14.0%, significantly poorer than those with metastases in other lesions (43.7%). These studies indicated that a subdivision of the M1 stage for metastatic NPC could be essential to assist physicians in evaluating the prognostication and arranging the therapy. In the current study, the M1 stage subdivision was classified into three groups referring to the retrospective study reported by Shen et al. (13): M1a, single metastasis other than the liver; M1b, single hepatic metastasis, and/or multiple metastases other than the liver; and M1c, multiple hepatic metastases. The 5-year OS rates for M1a, M1b and M1c were 62.6%,40.4% and 0%, respectively. Zou et al. (4) divided the M1 stage into three categories: M1a, oligo-metastasis in any location excluding the liver; M1b, multiple metastases except the liver; M1c, liver involvement regardless of metastatic lesions. They founded that the M1a and M1b classification of NPC may benefit from aggressive treatment, while chemoradiotherapy did not benefit patients in M1c. Owing to the limitation of the number of cases, we did not analyze the treatment strategies of patients classified into different subgroups. More prospective randomized controlled trials are needed to validate the new M1 stage subdivision, in order to assist physicians in determining the most satisfactory medical care for metastatic NPC patients.

Another prognostic factor recognized in the current study was pre-treatment serum LDH level. It has been reported that elevated levels of LDH may be relevant to large tumor burden, high risk of metastasis, and extension of tumor growth (27–29). In the study of Zeng et al. (8), the 3-year OS rate of patients with LDH ≤ 245U/L was 47.7%, remarkably better than those with LDH>245U/L(13.7%). Furthermore, the increase of LDH levels had been seen in no less than 60% of patients with hepatic metastasis. In the current study, the 5-year OS rate of patients with pre-treatment serum LDH≤ 180 U/L was 60.9%, compared with LDH>180U/L with a 5-year OS rate of 35.2% (HR 1.931, 95% CI 1.134-3.287, P = 0.015). On the basis of these results, we considered that pre-treatment serum LDH level may be a potential predictive indicator.

In summary, we conducted a retrospective study to evaluate the benefits of locoregional IMRT combined with chemotherapy in patients with metastatic NPC and found that chemotherapy cycles, pre-treatment serum LDH, M1 stage subdivision were independent prognostic indicators of survival for NPC patients with distant metastasis at initial diagnosis. The median overall survival time was 54.2 months, and the 5-year overall survival rate was 47.0% (total of 129 patients). The treatment efficacy was obviously better than the results reported previously. The potential explanations for the high level of long-term survival rate were as follows: 1. All the patients received IMRT, the improved target coverage afforded by IMRT can result in higher rates of local control; 2. All the patients underwent platinum-based systemic chemotherapy, which may lead to promising efficacy and tolerable toxicity; 3. Due to the limited number of cases, random errors cannot be ignored.

However, several deficiencies in the current study should be recognized. Firstly, this study was accomplished in one single medical establishment, which may influence the generality of our study and result in the limited sample size. Secondly, the treatment modalities were always individualized at the discretion of the clinicians and wishes of the patient, and thus, the impressive survival in our study and the analysis of prognostic factors might be impacted by a possible selection bias. Finally, the circulating EBV DNA load has been indicated as a tumor marker for disseminated NPC (30). Owing to the shortage of EBV DNA data in more than half of these patients, we excluded the factor to avoid the potential bias.

## Conclusion

In conclusion, patients with synchronous metastatic nasopharyngeal carcinoma may obtain long-term survival after active treatment. It is preliminarily believed that platinum-based induction chemotherapy combined with nasopharyngeal and cervical intensity-modulated radiation therapy can prolong survival. In the current study, we identified some negative prognostic factors which included chemotherapy cycles<4, pre-treatment serum LDH>180U/L, M1b (single hepatic metastasis and/or multiple metastases other than the liver), and M1c (Multiple hepatic metastases). It can be used in conjunction with the conventional TNM staging system for selecting the appropriate patients who may benefit from the combination of systemic chemotherapy and IMRT. Further well-designed prospective randomized studies are needed to optimize treatment strategies for NPC patients with synchronous metastasis.

## Data Availability Statement

The raw data supporting the conclusions of this article will be made available by the authors, without undue reservation.

## Ethics Statement

The studies involving human participants were reviewed and approved by Institutional Review Board of Fudan University Shanghai Cancer Center. The patients/participants provided their written informed consent to participate in this study.

## Author Contributions

MN, LG, FK, RZ, CD, and YL collected the data and finished the quality control of data. HY and CH provided the study concepts, designed the study, and performed statistical the analysis. MN, LG, HY, and CH wrote the manuscript. All authors contributed to the article and approved the submitted version.

## Conflict of Interest

The authors declare that the research was conducted in the absence of any commercial or financial relationships that could be construed as a potential conflict of interest.

## Abbreviations

AJCC, American Joint Committee on Cancer; CI, confidence interval; CTV, clinical target volume; GTV, gross tumor volume; Gy, Gray; HR, hazard ratio; IMRT, intensity-modulated radiotherapy; U, unit; L, liter; LDH, lactate dehydrogenase; MRI, magnetic resonance imaging; CT, computed tomography; NPC, nasopharyngeal carcinoma; OS, overall survival; PFS, progression-free survival; PTV, planning target volume; ROC, receiver operating characteristics; SLDH, serum lactate dehydrogenase; NCCN, National Comprehensive Cancer Network; TNM, tumor-node-metastasis.
